# Discordant multi-decadal trend in the intensity of the Kuroshio along its path during 1993–2013

**DOI:** 10.1038/s41598-018-32843-y

**Published:** 2018-10-02

**Authors:** You-Lin Wang, Chau-Ron Wu

**Affiliations:** Department of Earth Sciences, National Taiwan Normal University, Taipei, Taiwan

## Abstract

The Kuroshio transports warm water in the Pacific poleward from the tropics and plays a crucial role in modulating surrounding climate. Based on independent data sets, we demonstrated that the Kuroshio weakened downstream east of Taiwan, but intensified upstream east of Luzon Island during 1993–2013. The surface velocity (volume transport) of the Kuroshio has decreased 12.5% (4~5%) off east Taiwan but increased 18% (8~18%) off east Luzon. The discordant upstream–downstream trend was attributable to changes in oceanic eddies and basin surface winds: greater (lesser) cyclonic eddies, lesser (greater) anticyclonic eddies, and positive (negative) tendency in the Pacific Basin wind curl contributed to a weakened (intensified) downstream (upstream) Kuroshio. The difference in water mass between the upstream and downstream Kuroshio was balanced by an anomalous eastward flow, the southern branch of the Subtropical Counter Current which was enhanced and evacuated the redundant water eastward into the Pacific.

## Introduction

In the late 1990s, global climate shifted into a cold regime that ended in 2012–2013^1,2^. During this period, known as the “global warming hiatus,” the rate of global warming decreased^3,4^, which was shown to be correlated with the negative phase of the Pacific Decadal Oscillation (PDO)^5,6^, the Interdecadal Pacific Oscillation (IPO)^4,7^, or the El Niño–Southern Oscillation (ENSO)^3,8^. Synchronous changes in atmospheric and oceanic circulation during this period included intensified easterlies, which cooled the underlying sea surface temperature, moved warm water westward, and caused warming in the Pacific Warm Pool^3,4,9,10^. The warming signal of the Warm Pool was transported along the Kuroshio poleward into the marginal seas and extratropics^11^, and followed the Indonesian throughflow westward into the Indian Ocean^12^.

The Kuroshio, which forms the western part of the North Pacific subtropical gyre, serves as a conveyor moving heat and moisture from the tropics to the extratropics and modulating regional climate along its path^13,14^. On seasonal variability, the Kuroshio in the East China Sea (ECS) is weakened and onshore migration, while its width becomes wide during cold season. During warm season, on the other hand, the Kuroshio is strengthened with offshore migration, and its width is getting narrower^15^. The Kuroshio transport observed in the eastern Luzon has weakened (intensified) when the bifurcation latitude of the North Equatorial Current (NEC) has migrated northward (southward) and the NEC transport has weakened during cold (warm) season^16,17^. On interannual variability, fluctuations of the Kuroshio are correlated to various climate factors such as the ENSO and the PDO^13,18–21^. On multi-decadal timescales, the Kuroshio east of Taiwan (Luzon) is mainly governed by eddy activity (local wind forcing)^22^. Under 20^th^ century global warming, the poleward path shift of the Kuroshio moved more heat into its extension region^23^. During the two decades of the global warming hiatus, the Kuroshio weakened, but still transported more warm water poleward due to enhanced warming over the upstream heat source, the Pacific Warm Pool^11^. The supply of heat flux from the Pacific Warm Pool, combined with the intensity of the Kuroshio, affects the role of the Kuroshio in regional climate.

**Figure 1:**
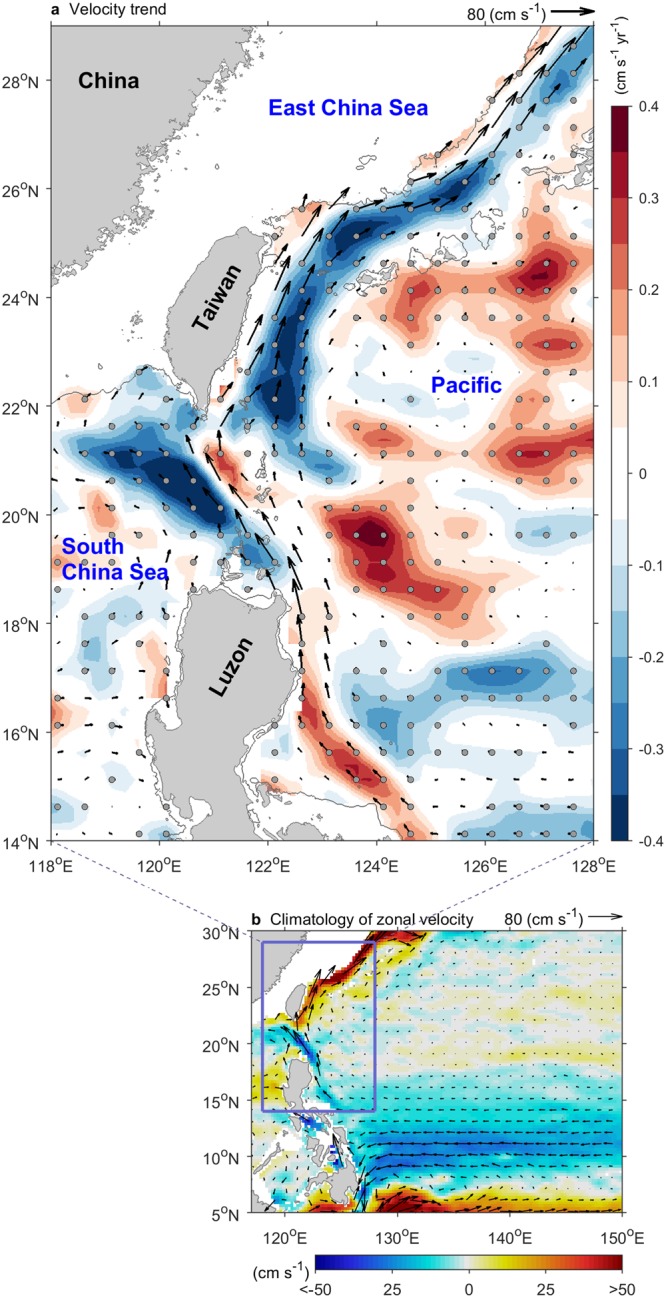
Decadal discordant trends along the Kuroshio during 1993–2013. (**a**) Mean speed (vector), linear trend (shading), statistical significance above the 99% confidence level (gray dots), 200 m isobaths (contour) are shown. (**b**) Mean zonal velocity (shading), mean speed (vector), and study area (box) are shown. Data shallower than 200 m depth are ignored. Data are provided from the AVISO (MADT two-sat version).

Figure 1a shows that the upstream Kuroshio east of Luzon Island intensified during 1993–2013, while the downstream Kuroshio east of Taiwan weakened. Between these regions lies the Luzon Strait, which is approximately 250 km wide. The opposite trend in the changes in intensity of the Kuroshio in the two locations implies the presence of a dynamic modulation in the Luzon Strait. The NEC bifurcation latitude tended to migrate southwards which resulted in the intensified upstream Kuroshio^10,24^. The Pacific Basin wind stress curl (WSC) and mesoscale eddy activity at these latitudes were primarily responsible for the discordant trend in Kuroshio intensity, and the Kuroshio water budget was balanced by an anomalous eastward flow in the area between the discordant two regions. In this paper, Section 2 describes the data sets and methods, Section 3 presents the effects of changes in basin winds and mesoscale eddies, and Section 4 discusses the Kuroshio water budget under the discordant trend along its path.

### Discordant multi-decadal trend along the Kuroshio

The AVISO product, spanned from 1993 to 2013, has generally been used to estimate the multi-decadal trend in oceanic surface velocity, as well as the sea surface height (SSH) response to the hiatus^4,9–11,24^. Figure 1a shows the mean state of surface velocity during 1993–2013 from the AVISO data. The Kuroshio is indicated as the fast velocity region from east of Luzon Island northward to east of Taiwan, and into the ECS. The upstream Kuroshio east of Luzon Island (14–18°N) intensified, which is consistent with the findings of previous studies^4,24^. The maximum increase in flow was 0.3 cm s^−1^ yr^−1^, located around 16°N (increased 18%; mean is 33 cm s^−1^). However, the downstream Kuroshio east of Taiwan (22–26°N) weakened and was two- to three-fold weaker than in the ECS (north of 26.5°N). The maximum decrease in flow was −0.5 cm s^−1^ yr^−1^ east of Taiwan (23°N) (decreased 12.5%; mean is 80 cm s^−1^), and −0.1 to −0.2 cm s^−1^ yr^−1^ in the ECS. The *in-situ* data from Argos drifter, and two validated high-resolution ocean reanalysis, HYCOM and JCOPE2, also support this discordant trend (see Supplementary Fig. S1).

Furthermore, in Supplementary Fig. S2, northward volume transports of the Kuroshio east of Luzon Island and east of Taiwan were calculated using the JCOPE2 and HYCOM reanalysis. Mean transports of the Kuroshio from HYCOM and JCOPE2 are comparable with historical measurement, as Table 1 in Hsin *et al*.^25^. During 1993–2013, the transport of the downstream Kuroshio east of Taiwan has decreased by about 4~5% (−0.07 Sv yr^−1^, mean is 31.5 Sv based on HYCOM; −0.07 Sv yr^−1^, mean is 28.1 Sv from JCOPE2), but increased by 8~18% in the upstream east of Luzon Island (+0.08 Sv yr^−1^, mean is 19.9 Sv from HYCOM; +0.11 Sv yr^−1^, mean is 12.5 Sv from JCOPE2). The discordant trend is not only visible in the surface Kuroshio but also confirmed in its volume transport. Sensitive tests demonstrate that the result is robust and not sensitive to the definition of the section (Fig. S3).

The distance between the two discordant regions is less than 300 km, and the discordant trend implies that the dynamic mechanisms differed between the two regions. In theory, negative WSC over the ocean basin produces a northward western boundary current; however, in reality, the Kuroshio is also modulated by mesoscale eddies^26–29^. Below, we discuss the relative contributions of the two factors.

### Wind effects

**Figure 2:**
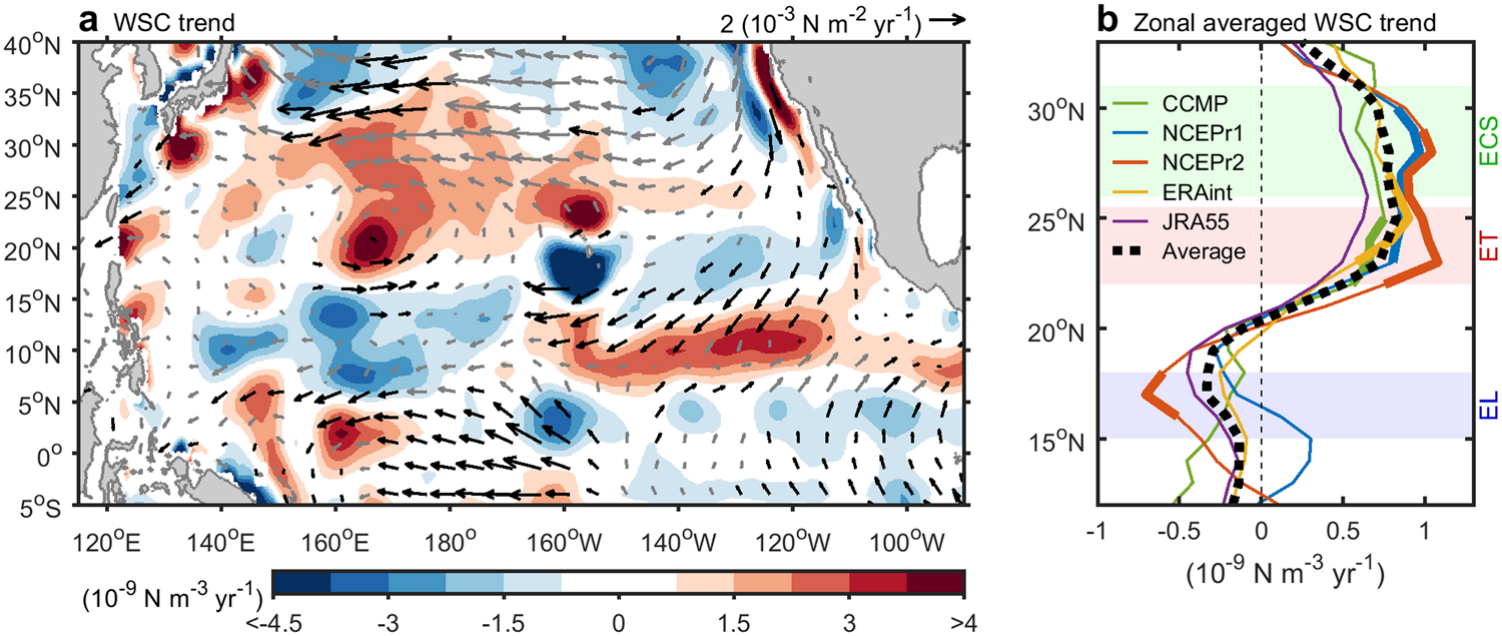
Linear trend of surface wind during 1993–2013. (**a**) The wind stress curl (WSC) trend (shading) and wind stress trend (vector). Data are provided from NCEPr2. Black (gray) vector indicates statistical significance above (below) the 90% confidence level. (**b**) Linear trends of zonal average of the Pacific basin WSC (122°E to western coast of the North America) from CCMP, NCEPr1, NCEPr2, ERAint, JRA55, and CCMP are shown. Latitudes of the East China Sea (ECS; green shading, 26–31°N), east of Taiwan (ET; red shading, 22–25.5°N), and east of Luzon (EL; blue shading, 15–18^o^N) are shown. Bold (thin) lines indicate statistically significant above (below) the 90% confidence level. Dash curve indicates the average of the five reanalysis products.

The mean state of Pacific surface winds as a combination of westerlies in the mid-latitudes and easterlies in low latitudes produces a negative basin-wide WSC that injects momentum into the Kuroshio (see Supplementary Fig. S4). During 1993–2013, based on the NECPr2 product, the westerlies (30–40°N) weakened, while the easterlies (south of 10°N) synchronously intensified (Fig. 2a). The multi-decadal trends in the two wind belts were associated with a regime shift in the PDO in 1999 from positive to negative phase^4,6,11,24^. Because a canonical negative phase of the PDO was correlated with weak westerlies and strong easterlies^5,30^, the overall pattern of the Pacific WSC multi-decadal trend was similar to that of the negative-phase PDO^11^.

The positive trend in the basin-wide WSC associated with the weakened westerlies was located in the subtropical latitudes of the downstream Kuroshio region (20–30°N; Fig. 2a). Two peak signals were observed at 160–165°E and 20–25°N, and north of Hawaii. The negative trend in the WSC associated with the intensified easterlies was located in the tropical latitudes (south of 20°N). Major negative signals were located at 170–175°E and 5–10°N, and south of Hawaii. The positive WSC trend over the subtropics and the negative WSC trend over the tropics contributed to the weakened downstream and intensified upstream of the Kuroshio, respectively, via the Sverdrup transport.

As shown in Fig. 2b, the trends of the Pacific basin WSC (zonal average from east of 122°E to the western coast of North America) based on various data sets, including the NCEPr1, NCEPr2, ERAint, JRA55, and CCMP, lend further support to the role of the WSC: the basin-wide WSC at latitudes of 22–32°N (red and green shading in Fig. 2b) tended to be positive, but it tended to be negative at latitudes south of 18°N (blue shading in Fig. 2b). In addition, the trends of the basin WSC in latitudes of the ECS, east of Taiwan, and east of Luzon Island have respectively decreased by 19% (+0.75 × 10^−9^ N m^−3^ yr^−1^, mean is −0.8 × 10^−7^ N m^−3^), decreased by 33% (+0.75 × 10^−9^ N m^−3^ yr^−1^, mean is −0.45 × 10^−7^ N m^−3^), and increased by 30% (−0.3 × 10^−9^ N m^−3^ yr^−1^, mean is −0.2 × 10^−7^ N m^−3^), compared with climatology in Supplementary Fig. S4. Furthermore, the general pattern of the basin-wide WSC tendency in the four reanalysis products was consistent with that derived from satellite observations, the CCMP product (see Supplementary Fig. S5). Results from various data sets in Fig. 2b confirmed that the strengthening WSC in the tropics and weakening WSC in the subtropics lend the increasing upstream Kuroshio transport but decreasing downstream Kuroshio transport.

The basin WSC induced-Sverdrup transport from various wind reanalysis is shown in Supplementary Fig. S6. The Sverdrup transport of the Kuroshio has decreased by about 28% north of Taiwan (−0.4 to −0.7 Sv yr^−1^, mean is 30~50 Sv), but increased by about 45% east of Luzon Island (+0.2 to +0.7 Sv yr^−1^, mean is about 20 Sv), compared with climatology in Supplementary Fig. S7. The trends of WSC and Sverdrup transport east of Taiwan (red shading in Fig. 2b) and in the ECS (green shading in Fig. 2b) were similar to one another, implying that the momentum injected from the basin-wide winds to the Kuroshio east of Taiwan and into the ECS was similar. Thus, the two- to three-fold weakening of the Kuroshio east of Taiwan relative to that in the ECS was not tied to basin-wide changes in wind, and requires a different explanation.

### Eddy effects

**Figure 3:**
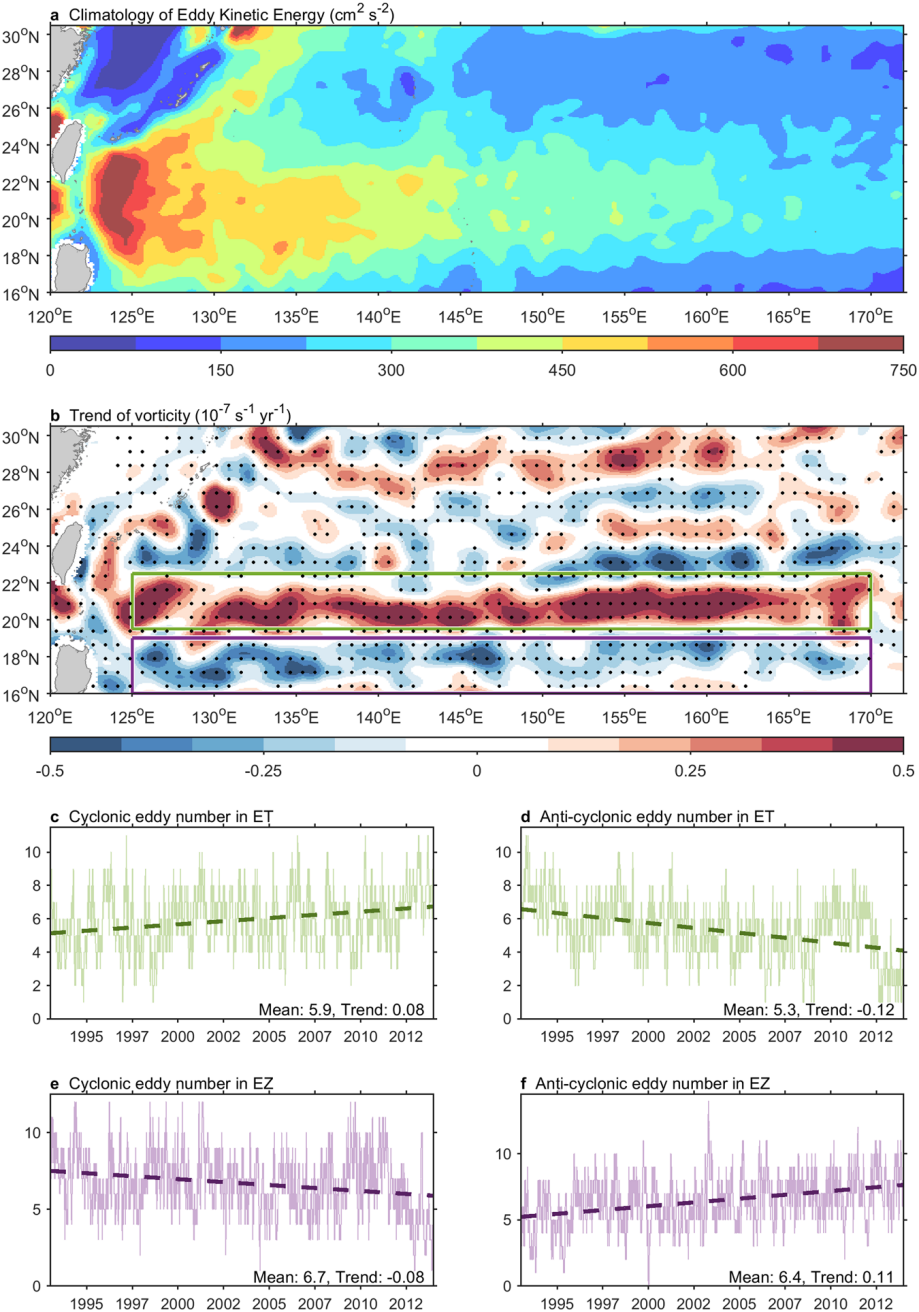
Linear trend of mesoscale eddy number during 1993–2013. (**a**) Climatology of Eddy Kinetic Energy (EKE). (**b**) Linear trend of vorticity. Gray dots indicate statistical significance above 99% confidence level. Green and purple boxes are the region of east Taiwan (ET; 19.5–22.5°N) and east Luzon (EL; 16–19°N), respectively. Spatial running average with window of 2° × 2° has been applied to emphasize the signal of mesoscale eddies. (**c**,**d**) Number of cyclonic and anticyclonic eddy in ET, respectively. (**e**,**f**) Number of cyclonic and anticyclonic eddy in EL, respectively. All of trends are statistical significance above the 99% confidence level. Data are provided from the AVISO (the MSLA two-sat version used in (**a**,**b**) the META product used in (**c**–**f**)). The counting of eddies is defined as total number of eddies existed in the box each day.

The Kuroshio east of Taiwan frequently collides with westward-propagating oceanic mesoscale eddies generated in the Subtropical Counter Current (STCC), which zonal eastward current located in the band of 18–25°N^27^. The surface eastward STCC interacts with the subsurface westward NEC, producing a vertical shear that favors the generation of mesoscale eddies (Fig. 3a). As the two currents are modulated by changing atmospheric circulation, eddy activity can vary on interannual and decadal timescales^27,31^. The Kuroshio intensity becomes weaker (stronger) when a cyclonic (anticyclonic) eddy collides with it^26,27,29^, implying that the modulation of Kuroshio intensity was associated with changing mesoscale eddies during 1993–2013.

During 1993–2013, vorticity trend eastward along the STCC was generally positive (green box in Fig. 3b), which implies that cyclonic eddies were more active than anticyclonic eddies, contributing to downstream Kuroshio weakening east of Taiwan. In contrast, vorticity trend off the eastern Luzon was generally negative (purple box in Fig. 3b), which implies an increase in anticyclonic eddy activity relative to cyclonic activity, contributing to upstream Kuroshio strengthening east of Luzon Island. Furthermore, no clear trend in vorticity was observed at the latitudes spanned by the ECS (25–30°N in Fig. 3b), indicating that the multi-decadal change in eddy activity generated at the latitude was less effective in modulating the Kuroshio than was that generated east of Taiwan. Overall, cyclonic eddies tended to dominate, contributing to an enhancement of the weakening of the Kuroshio east of Taiwan relative to that in the ECS, whereas anticyclonic eddies tended to be dominant in the upstream Kuroshio east of Luzon Island, contributing to an intensified upstream flow.

The positive multi-decadal trend in vorticity implies the presence of more cyclonic eddies and/or fewer anticyclonic eddies. Statistical datasets of eddy properties, the Mesoscale Eddy Trajectory Atlas (META), were used to elucidate this relationship. In Fig. 3c, the number of cyclonic eddies in the STCC region (19.5–22.5°N; green box in Fig. 3b) has increased by 27% (+0.08 yr^−1^, mean is 5.9) during 1993–2013. Synchronously, the number of anticyclonic eddies has decreased by 45% (−0.12 yr^−1^, mean is 5.3) (Fig. 3d). Both results of increasing cyclonic eddies and decreasing anticyclonic eddies along the STCC lend the positive trend of vorticity (green box in Fig. 3b) and the weakening Kuroshio east of Taiwan (Fig. 1).

In Fig. 3e, the number of cyclonic eddies east of Luzon Island (16–19°N; purple box in Fig. 3b) decreased by 24% (−0.08 yr^−1^, mean is 6.7), while anticyclonic eddies increased by 34% (+0.11 yr^−1^, mean is 6.4) during 1993–2013 (Fig. 3f). Decreasing cyclonic eddies and increasing anticyclonic eddies confirm the negative trend of vorticity (purple box in Fig. 3b) along the STCC and the strengthening Kuroshio east of Luzon Island (Fig. 1). The results shown in Fig. 3 support the hypothesis that changes in eddy activity contributed to the discordant trends.

## Discussion and Conclusion

During 1993–2013, multi-decadal discordant trends in surface velocity occurred along the Kuroshio (intensified upstream and weakened downstream), which were tied to changes in the Pacific basin WSC and mesoscale eddies: off east Taiwan (east Luzon), the weakened (intensified) Kuroshio during 1993–2013 was attributed to the positive (negative) WSC trend and increased (decreased) ratio of cyclonic/anticyclonic eddies in the latitude of eastern Taiwan (eastern Luzon). Recent advance led further support that the eddy activity is the major factor governing the Kuroshio east of Taiwan, and the local wind effect mainly modulates the Kuroshio in the Luzon Strait and east of Luzon on the multi-decadal timescale^22^. The Kuroshio acts as a conduit to transport warm water poleward, and the discordant trends affected this transport. How was the Kuroshio water budget conserved during this period? Additional water flux from increased upstream and weakened downstream flow required balancing along the sides of the Kuroshio.

**Figure 4:**
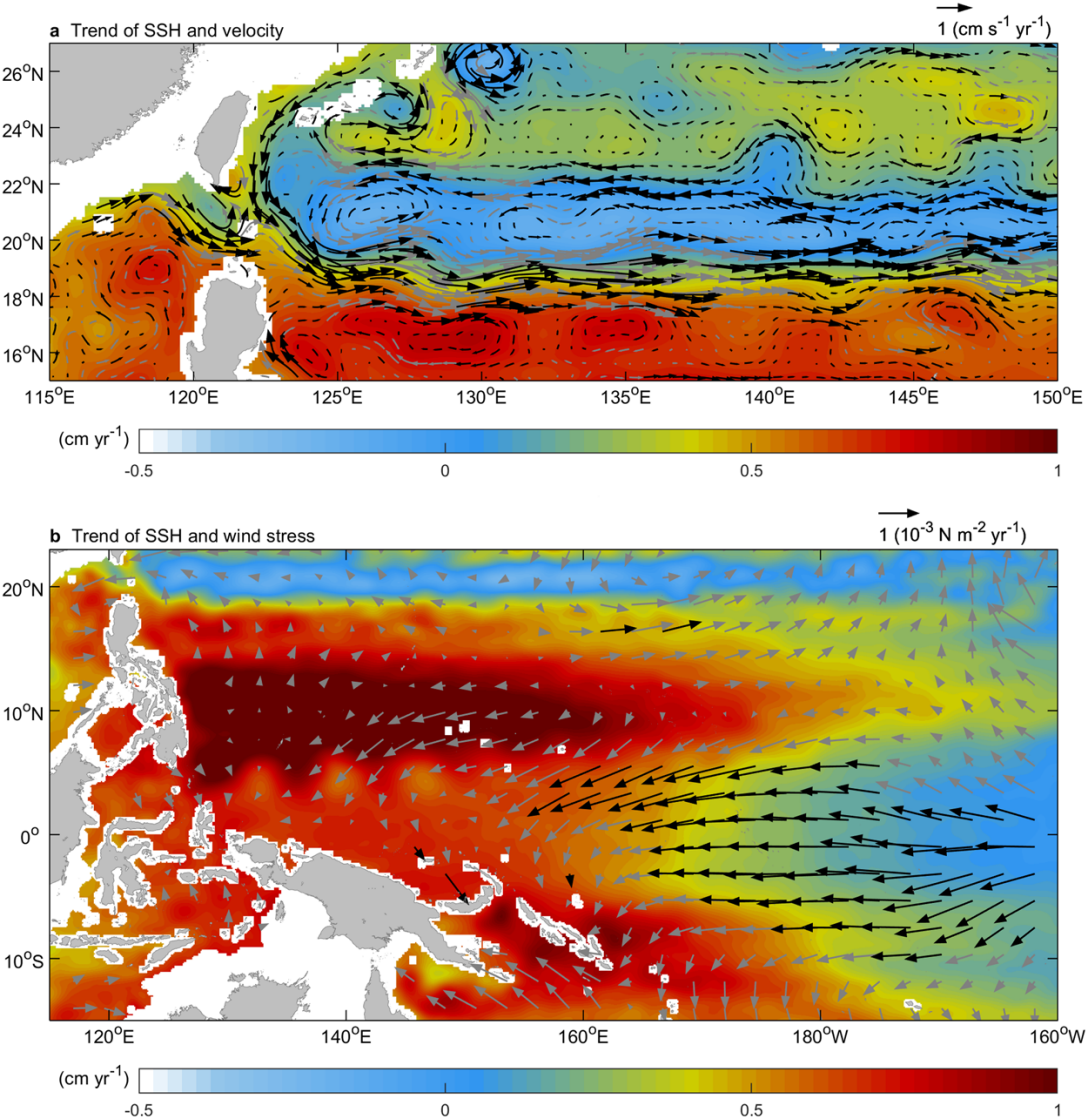
Strengthening southern branch of the Subtropical Counter Current during 1993–2013. (**a**) Linear trend of sea surface height (SSH; shading) and geostrophic velocity (vector). Data are provided by the AVISO (MADT two-sat version). (**b**) Linear trend of SSH (shading) and wind stress (vector) over the western North Pacific warm pool. Black (gray) vector is statistical significance above (below) the 99% confidence level. Data shallower than 200 m depth are ignored.

Figure 4a shows the velocity in the discordant trend regions. The northward trend (vector) east of Luzon Island and the southward trend east of Taiwan are shown as the discordant trends. A pronounced eastward trend in velocity was observed from the northern South China Sea (SCS) into the west side of the Kuroshio (black vector at 120°E and 20°N), which is a well-known region of the Kuroshio where it intrudes into the SCS^32–36^. The eastward trend indicates that the Kuroshio intrusion into the SCS weakened, consistent with the results of recent studies^21,24,37^. Previous studies suggested that the weakened intrusion can be attributed to the intensified Kuroshio off east Luzon via dominated northward inertia^11,21,24,38,39^. The eastward velocity trend provided additional water input into the Kuroshio; hence, the lost Kuroshio water from the discordant trend was not compensated for by water flux from the western side of the Kuroshio. In fact, redundant water was evacuated via the strengthening southern branch of the STCC (S-STCC) formed southeast of Taiwan (122.5°E and 22°N), flowing eastward into the Pacific (eastward vector at 17–21°N in Fig. 4a).

As a geostrophic current associated with a sea-level gradient across it (Fig. 4a), the strengthening eastward S-STCC was tied to the combination effect between mesoscale SSH trend of eddies and basin-scale SSH trend over the Pacific warm pool. In Fig. 3, the increasing number of cyclonic eddies and decreasing number of anticyclonic eddies were capable of contributing the SSH dropping along the north-side of the strengthening S-STCC, while the rising SSH over the warm pool (Fig. 4b) increased the SSH along the south-side of the S-STCC, resulting in a northward pressure gradient force anomaly which enhanced the S-STCC. The increase in SSH in the warm pool was a response to an intensified trade wind (black vectors in Fig. 4b).

**Figure 5:**
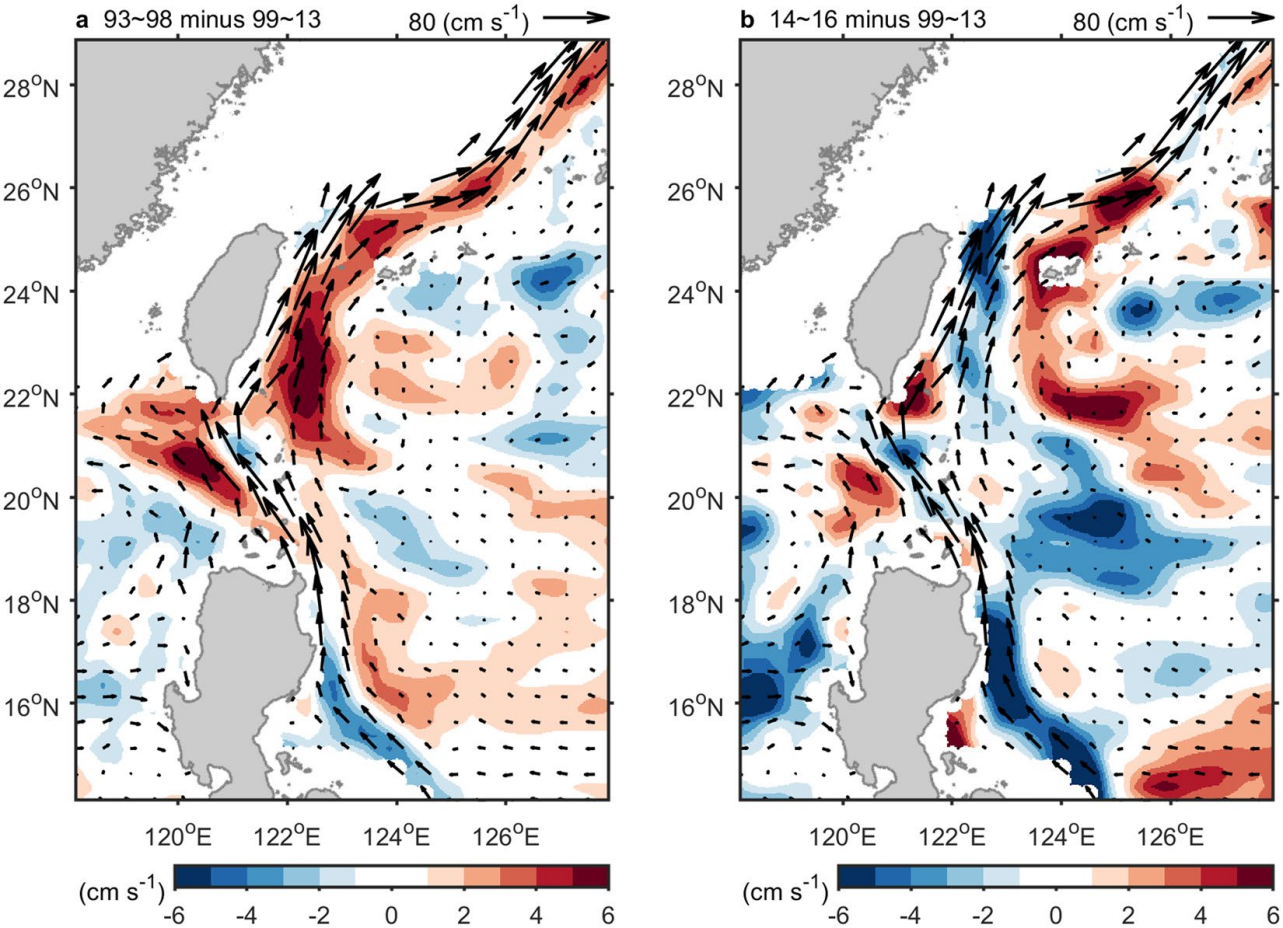
The Kuroshio during the two warming periods, before 1999 and after 2014. (**a**) Difference of velocity (AVISO MADT-two-sat version) via 1993~1998 minus 1999~2013. (**b**) Same as (**a**), but for 2014~2016 minus 1999~2013. Vector indicates climatology of velocity (1993–2016). Data shallower than 200 m depth are ignored.

Since the hiatus was ended at 2012–2013, the global climate became warming again. Does the Kuroshio during the two warming periods, before 1999 and after 2014, exist a consistency response? The answer is no. In Fig. 5, in comparison with the period of 1999–2013, the Kuroshio off east Luzon was weakened during both the first (1993~1998) and second (2014~2016) warming period. On the other hand, the Kuroshio off east Taiwan in the second warming period was weakened (Fig. 5b) which was distinct from the first warming period (Fig. 5a). The significant weakened Kuroshio during the second period may result from the 2014~2016 extreme El Niño. A wealth of studies demonstrated that the Kurushio became weakened when an El Niño event occurred^13^.

In summary, the Kuroshio multi-decadal discordant trend shared numerous traits between the intensified upstream and weakened downstream during 1993–2013. We identified two main factors in the discordant trend. (1) Increased activity of cyclonic eddies and decreased activity of anticyclonic eddies contributed to a weakened downstream Kuroshio east of Taiwan. In contrast, increased activity of anticyclonic eddies and decreased activity of cyclonic eddies contributed to an intensified upstream Kuroshio east of Luzon Island. (2) A positive (negative) trend in the Pacific WSC north (south) of 20°N weakened (intensified) the downstream (upstream) Kuroshio east of Taiwan (east of Luzon Island). The unbalanced water budget between the upstream and downstream compensated for by the strengthening eastward S-STCC in the region between the discordant regions. The decreasing SSH resulted from mesoscale eddies and the increase in SSH in the Pacific warm pool produced a northward pressure gradient during 1993–2013, which contributed to the strengthening eastward S-STCC.

## Methods

Four oceanic velocity data sets are used: geostrophic velocity data are provided by the AVISO (Archiving, Validation and Interpretation of Satellite Oceanographic data) (DT-MADT two-sat version; https://www.aviso.altimetry.fr), and the temporal and spatial resolution are daily and 1/4 degree, respectively. Argos drifter array data are provided by the GDP (Global Drifter Program) (http://www.aoml.noaa.gov/phod/dac/), and the temporal resolution is 6 hour. After downloaded, Argos data have been gridded into monthly–0.5 degree meshes. HYCOM + NCODA (Hybrid Coordinate Ocean Model + Navy Coupled Ocean Data Assimilation) ocean reanalysis data run daily at the Navy DoD Supercomputing Resource Center and are download from https://hycom.org (GLBu0.08 version), with the temporal and spatial resolution are daily and 1/10 degree, respectively. JCOPE2 (Japan Coastal Ocean Predictability Experiment 2) ocean reanalysis data are provided by the JAMSTEC (Japan Agency for Marine-Earth Science and Technology; http://www.jamstec.go.jp), and the temporal and spatial resolution are daily and 1/12 degree, respectively.

Four surface-wind stress data sets are used. The National Centers for Environmental Prediction (NCEP)/National Center for Atmospheric Research Reanalysis 1 (NCEPr1) data and NCEP–Department of Energy Atmospheric Model Intercomparison Project II Reanalysis (NCEPr2) data are downloaded from the National Oceanic and Atmospheric Administration/Earth System Research Laboratory website (www.esrl.noaa.gov). European Center for Medium-Range Weather Forecasts (ECMWF) Reanalysis Interim (ERAint) data are downloaded from the ECMWF website (https://www.ecmwf.int/). Japanese 55-year Reanalysis (JRA55) data are downloaded from the Japan Meteorological Agency website (http://www.jma.go.jp). The spatial resolution of the NCEPr1, NCEPr2, ERAint, and JRA55 is respectively 1.875° × 1.875°, 1.875° × 1.875°, 1° × 1°, and 1.25° × 1.25°. Those reanalysis products reveal a similar pattern of linear trend of surface wind to that derived from satellite observations, the CCMP (Cross-Calibrated Multi-Platform, downloaded from the Jet Propulsion Laboratory website, https://podaac.jpl.nasa.gov/). The spatial resolution of the CCMP product is 0.25° × 0.25°.

Sea level anomaly (SLA), SSH, and the Mesoscale Eddy Trajectory Atlas (META) products are also provided by the AVISO. The SLA data are using version of DT-MSLA two-sat, and the temporal and spatial resolution are daily and 1/4 degree, respectively. The SSH data are using version of DT-MADT two-sat, and the temporal and spatial resolution are daily and 1/4 degree, respectively. The META products are produced by SSALTO/DUACS and distributed by the AVISO, and the temporal resolution is daily. The statistical cyclonic-type data from META indicate the cyclonic and anticyclonic ocean eddies by labeled +1 and −1, respectively. Each eddy detected is based on the basis of connected pixels from daily DT-MSLA gridded product of the AVISO. The cyclonic-type data have been gridded into daily and 1 degree meshes after downloaded. The significance test of trend was performed based on the *f*-test. The Eddy Kinetic Energy is obtained based on geostrophic calculation^40^.

## Electronic supplementary material

Supplementray Information
