# Supplementary material for: Discordant multi-decadal trend in the intensity of the Kuroshio along its path during 1993–2013
Source: Sci Rep. 2018 Oct 2;8:14633. doi: 10.1038/s41598-018-32843-y (PMC6168475; doi:10.1038/s41598-018-32843-y)
Supplement: Supplementary file 1 — Supplementray Information [file 41598_2018_32843_MOESM1_ESM.docx]

**Supplementary Information**

**Title: Discordant multi-decadal trend in the intensity of the Kuroshio along its path during 1993-2013**

Authors: You-Lin Wang^1^ and Chau-Ron Wu^1,*^

*^1^Department of Earth Sciences, National Taiwan Normal University*

*^*^Correspondence to cwu@ntnu.edu.tw*


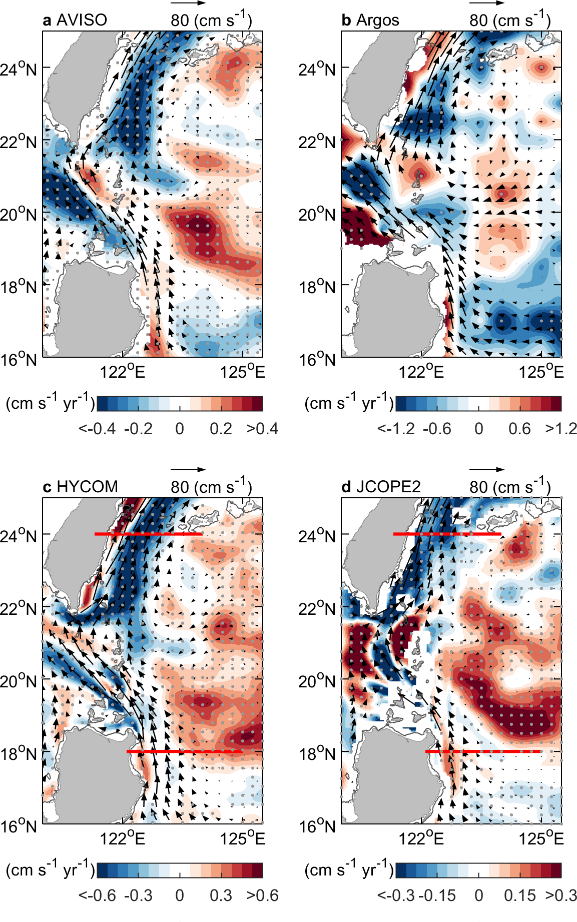


**Supplementary Figure S1.** Multi-decadal discordant trends between the Kuroshio off east Taiwan and off east Luzon from various data sets during 1993-2013. Mean velocity (vector), trend of speed (shading), statistical significance above the 99% confidence level (gray dots), and 200 m isobaths (contour) are shown. (c and d) Red lines indicate sections off east Taiwan (24°N) and east Luzon (18°N). Data shallower than 200 m depth are ignored. Depth in Figure S1b-S1d is 15 m depth.


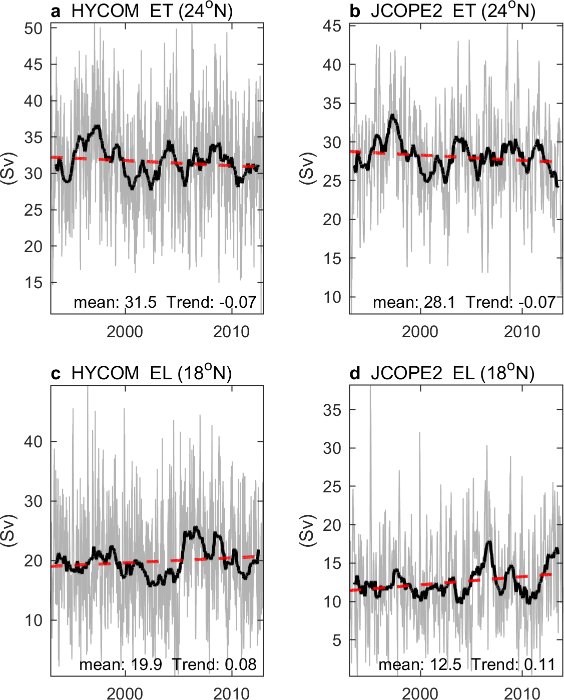


**Supplementary Figure 2.** Multi-decadal discordant trends of the Kuroshio northward transport (0-1000 m) in the eastern Taiwan (24°N) and eastern Luzon (18°N) (red lines in Supplementary Figure 1) during 1993-2013. Original daily data (gray line), 1-year running average (black line), and trend (red line) are shown. Value of mean transport and trend are shown in the bottom, and all trends are statistical significance above the 99% confidence level.


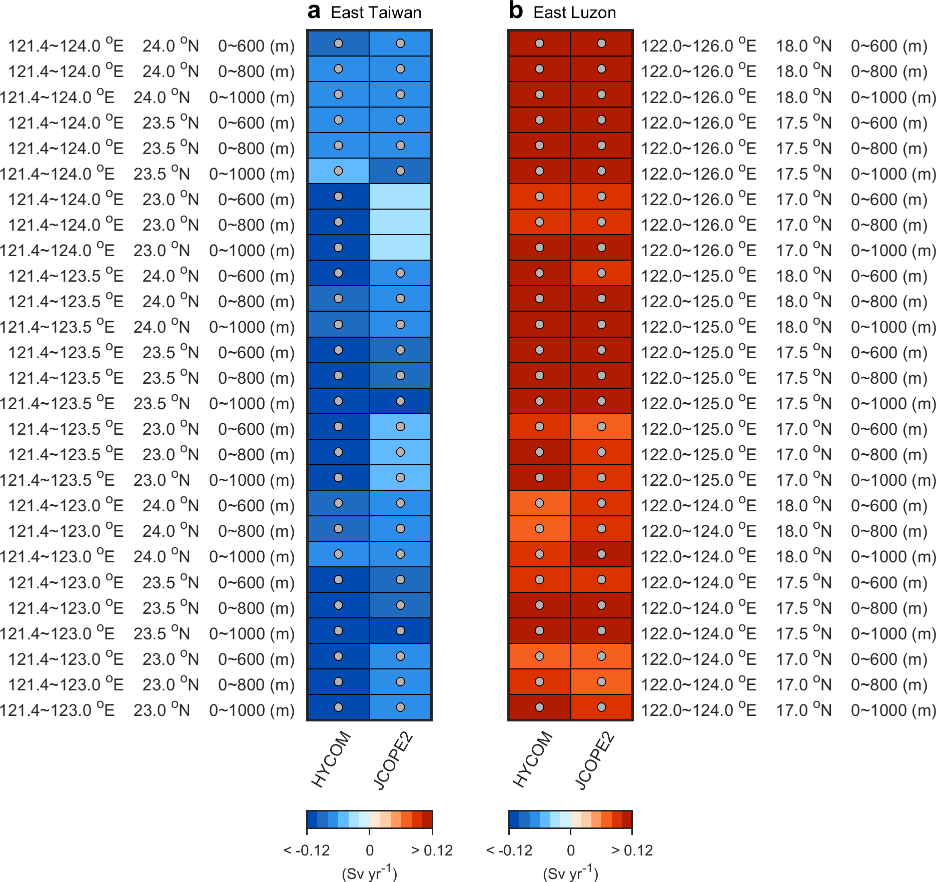


**Supplementary Figure S3.** Northward transport of the Kuroshio in the eastern Taiwan (a) and eastern Luzon (b), respectively. Each label along the Y-axis indicates definition of individual section (range of longitude, latitude, and range of depth) used to calculate the transport, label of the X-axis indicates various data sets used. Gray dot indicates the statistical confidence above the 99% significance level.


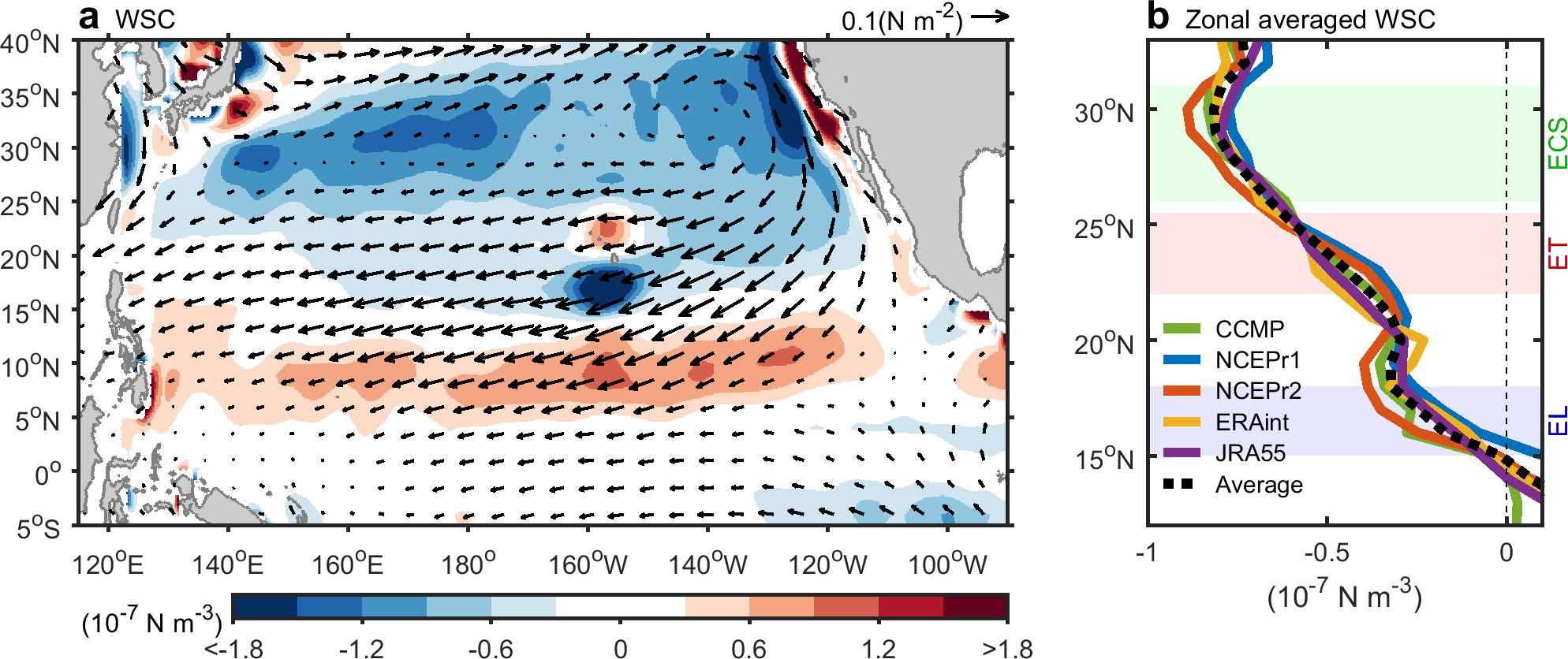


**Supplementary Figure S4.** Mean state of surface wind during 1993-2013. (a) Mean wind stress (vector) and mean wind stress curl (WSC) (shading) from the NCEPr2. (b) Pacific basin zonal average of WSC (east 122°E to the western coast of the North America) from various data sets. Dash curve indicates the average of the four reanalysis products.


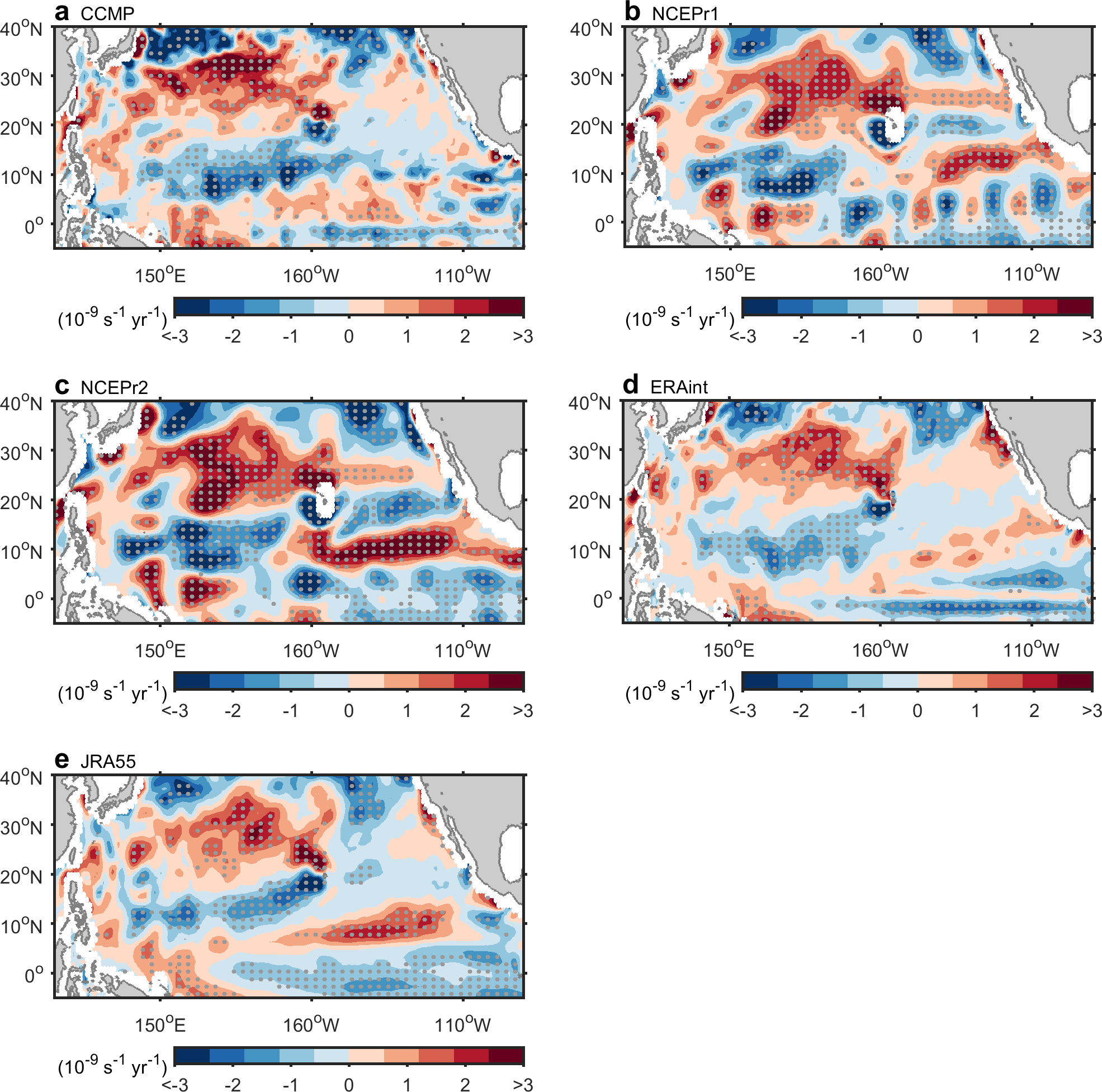


**Supplementary Figure S5.** Linear trend of wind curl during 1993-2013 from various data sets. Dots indicate statistical significance above 90% confidence level.


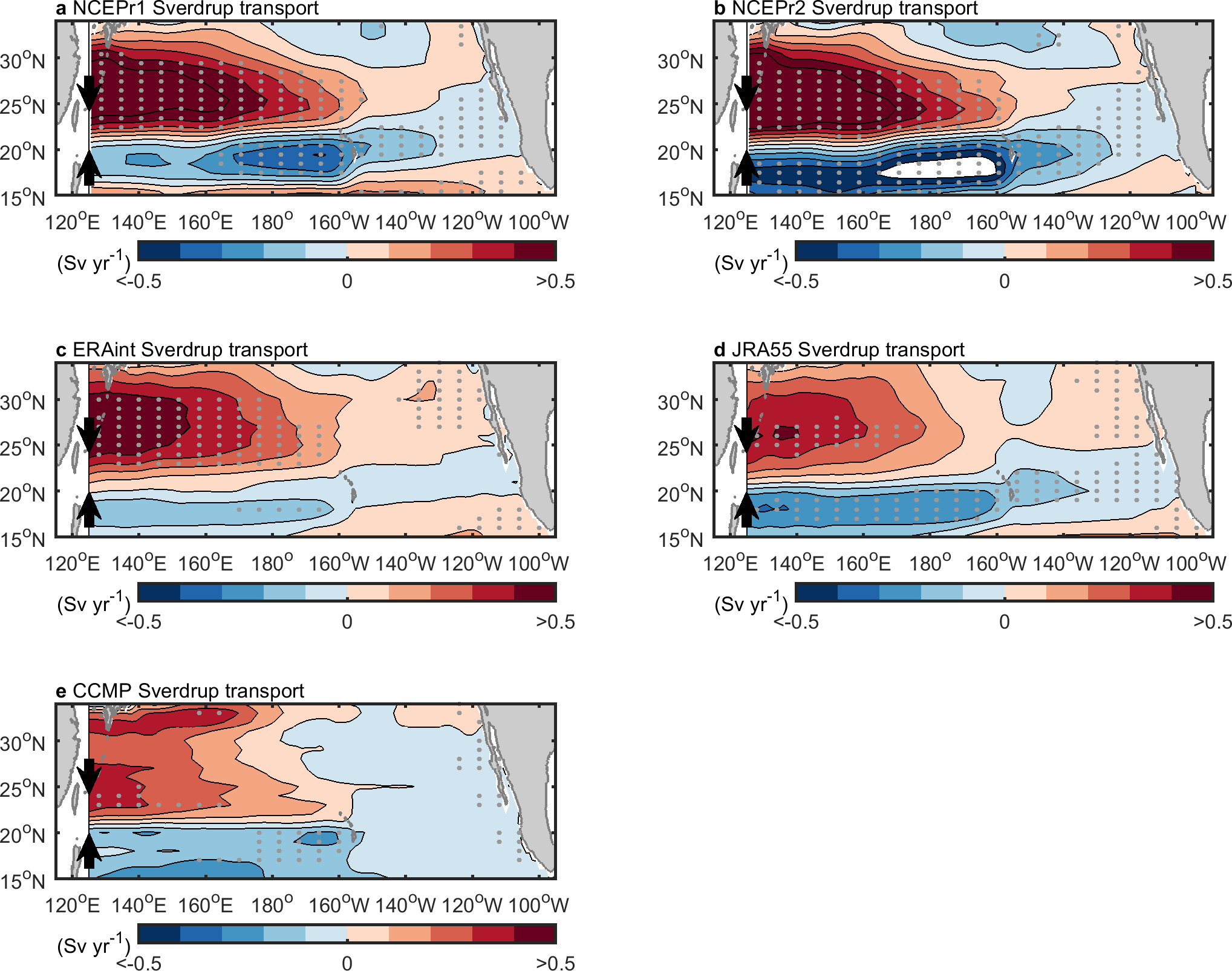


**Supplementary Figure S6.** Linear trend of wind-induced Sverdrup transport (shading and contour) from various data sets during 1993-2013. Gray dots indicate statistical significance above the 90% confidence level. Vector indicates direction of the Kuroshio transport trend. Interval of contour is 0.1 Sv yr^-1^.


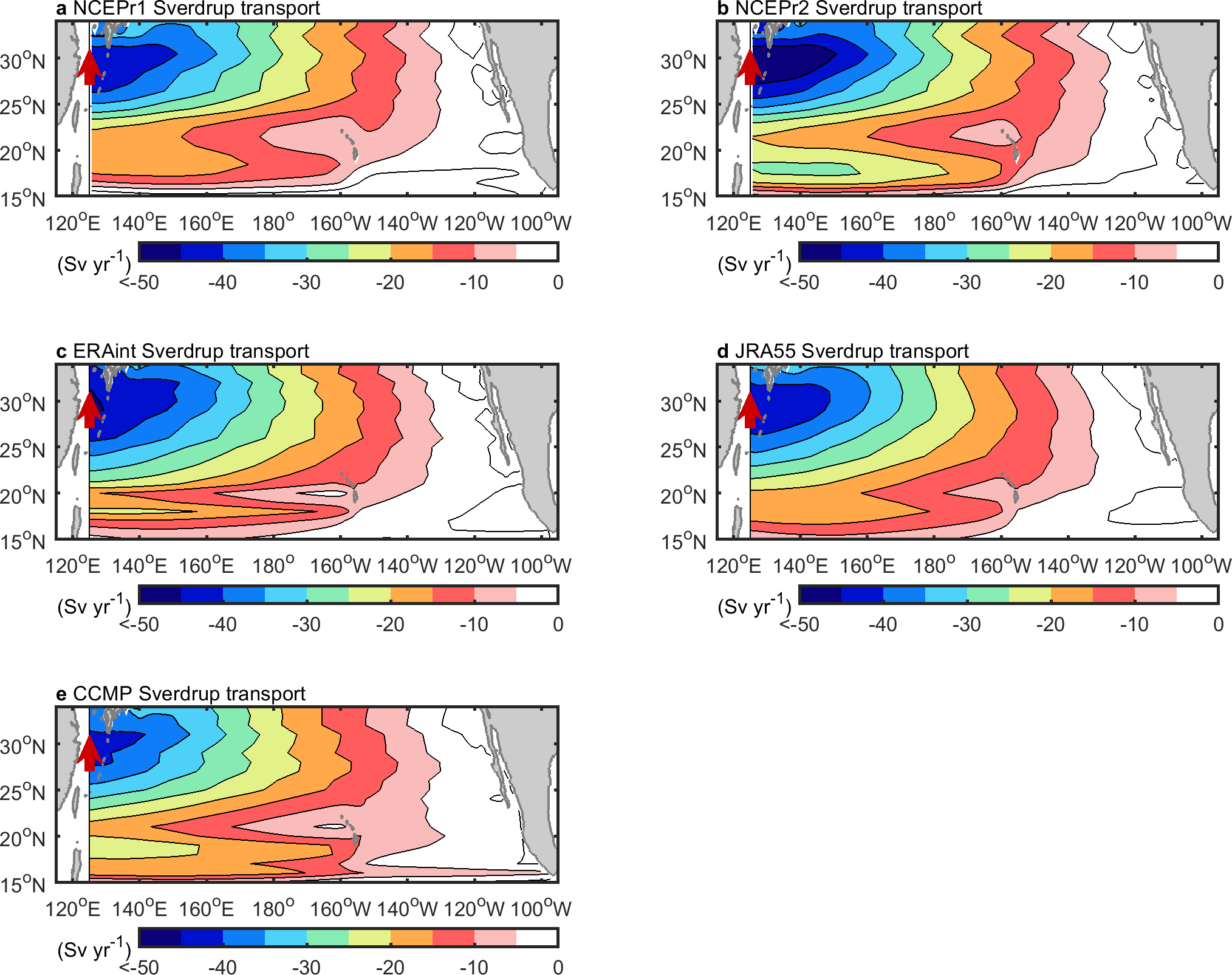


**Supplementary Figure S7.** Mean state of wind-induced Sverdrup transport (shading and contour) from various data sets during 1993-2013. Vector indicates direction of the Kuroshio. Interval of contour is 5 Sv.
